# Activation of c-MET Induces a Stem-Like Phenotype in Human Prostate Cancer

**DOI:** 10.1371/journal.pone.0026753

**Published:** 2011-11-14

**Authors:** Geert J. L. H. van Leenders, Rajesh Sookhlall, Wilma J. Teubel, Corrina M. A. de Ridder, Suzanne Reneman, Andrea Sacchetti, Kees J. Vissers, Wytske van Weerden, Guido Jenster

**Affiliations:** 1 Department of Pathology, Erasmus Medical Center, Rotterdam, The Netherlands; 2 Department of Urology, Erasmus Medical Center, Rotterdam, The Netherlands; Wayne State University School of Medicine, United States of America

## Abstract

Prostate cancer consists of secretory cells and a population of immature cells. The function of immature cells and their mutual relation with secretory cells are still poorly understood. Immature cells either have a hierarchical relation to secretory cells (stem cell model) or represent an inducible population emerging upon appropriate stimulation of differentiated cells. Hepatocyte Growth Factor (HGF) receptor c-MET is specifically expressed in immature prostate cells. Our objective is to determine the role of immature cells in prostate cancer by analysis of the HGF/c-MET pathway.

Gene-expression profiling of DU145 prostate cancer cells stimulated with HGF revealed induction of a molecular signature associated with stem cells, characterized by up-regulation of CD49b, CD49f, CD44 and SOX9, and down-regulation of CD24 (‘stem-like signature’). We confirmed the acquisition of a stem-like phenotype by quantitative PCR, FACS analysis and Western blotting. Further, HGF led to activation of the stem cell related Notch pathway by up-regulation of its ligands Jagged-1 and Delta-like 4. Small molecules SU11274 and PHA665752 targeting c-MET activity were both able to block the molecular and biologic effects of HGF. Knock-down of c-MET by shRNA infection resulted in significant reduction and delay of orthotopic tumour-formation in male NMRI mice. Immunohistochemical analysis in prostatectomies revealed significant enrichment of c-MET positive cells at the invasive front, and demonstrated co-expression of c-MET with stem-like markers CD49b and CD49f.

In conclusion, activation of c-MET in prostate cancer cells induced a stem-like phenotype, indicating a dynamic relation between differentiated and stem-like cells in this malignancy. Its mediation of efficient tumour-formation *in vivo* and predominant receptor expression at the invasive front implicate that c-MET regulates tumour infiltration in surrounding tissues putatively by acquisition of a stem-like phenotype.

## Introduction

Within the prostate epithelium, tissue homeostasis is mediated by stem cells residing in the basal glandular epithelium [1]. After asymmetric division stem cells give rise to transit-amplifying cells, which are present in both basal and luminal epithelium, and which finally differentiate into luminal secretory cells. Various membranous markers are differentially expressed in stem and differentiated cells in benign rodent and human prostate epithelium including Sca-1^+^, α_6_-integrin/CD49f^+^, α_2_-integrin/CD49b^+^, CD133^+^, CD117^+^, CD44^+^ and CD24^−^ [2]–[9]. Combination of these markers might further delimitate stem, transit-amplifying and terminally differentiated cells in normal epithelium. For instance, stem cells putatively express α_2_β_1_-integrin^+^/CD133^+^, transit-amplifying cells are α_2_β_1_-integrin^+^/CD133^−^, and terminally differentiated cells are α_2_β_1_-integrin^−^/CD133^−^ [3], [7].

Cell populations with biological features resembling those of benign stem cells have also been identified in malignant tumours [10]–[13]. In prostate cancer, α_2_β_1_-integrin^+^/CD133^+^ cells possess potency for self-renewal and multi-directional differentiation *in vitro* [8], [14]. In addition, CD44^+^/CD24^−^ cells isolated from prostate cancer cell lines demonstrate high tumour-forming potential *in vivo* [4]. In spite of their apparent variability in clonogenic and tumour-initiating potential, the mutual relation between immature and differentiated cells is still poorly understood. In correspondence to their relation in normal tissues, a strict hierarchic relation between so-called cancer stem cells (CSC's) and differentiated cells has been postulated [12]–[14]. According to this model, CSC's are direct and irreversible progenitors of differentiated cells. Recently, however, it was demonstrated that differentiated cells can acquire CSC features in mammary and colon cancer [15], [16]. Particularly, phenotypic and biological characteristics contributed to stem cells can be gained, when more differentiated cells undergo epithelial-mesenchymal transition (EMT) either by forced depression of E-cadherin or by factors secreted by the micro-environment such as Hepatocyte Growth Factor (HGF) [15], [16]. Since its exact nature and relation with other cell types are still controversial, we refer to the cell population displaying stem cell characteristics as stem-like cells.

HGF and its tyrosine kinase receptor c-MET are important mediators of organogenesis, tissue regeneration and wound healing [17]. Within the normal prostate epithelium, c-MET is specifically expressed in basal and atrophic luminal cells, where it putatively mediates regeneration of damaged secretory glands [18], [19]. In prostate cancer, c-MET is present at low levels, with a minority of cells displaying high protein expression [18], [20], [21]. Previously, others and we have shown that c-MET and basal cell marker Keratin 5 are co-expressed within the same cell population in prostate cancer [14], [18]. Since the HGF/c-MET pathway has a regulatory function in migration and invasion *in vitro*, we have suggested that this cell population is specifically prone to infiltration in surrounding tissues [18], [22].

Little is known on the role of c-MET in relation to stem-like cells in prostate cancer and how *in vitro* studies on stem-like cells translate to actual cancer in patients. In this study, we demonstrate that activation of c-MET leads to induction of a stem-like phenotype in prostate cancer. Knock-down of c-MET further strongly reduced tumour-formation in nude mice. Finally, we demonstrate that c-MET is preferentially expressed at the perimeter of human prostatectomy specimens and is co-localized with its down-stream targets α_2_-integrin and α_6_-integrin. These data indicate that stem-like cells mediate tumour expansion at the invasive front of prostate cancer.

## Materials and Methods

### Ethics statement

The study was approved by the national Animal Experiment Committee (DEC; 102-08-01; EUR1396). All patient samples were anonymously used after appropriate written informed consent and under approval of the human ethics Institutional Review Board (METC; MEC02.0957).

### Cells and materials

DU145 prostate cancer cell line and human embryonic kidney (HEK) 293T cells were purchased from the American Type Culture Collection (ATCC, Rockville, MD, USA). DU145 was maintained at 37°C/5% CO_2_ in RPMI 1640 containing 5% fetal calf serum (FCS) and penicillin/streptomycin (P/S) (Lonza, Verviers, Belgium). For stimulation experiments, DU145 cells were seeded overnight in RPMI/FCS medium. After 1 day, medium was replaced by 5% Dextran Charcoal (DCC) treated RPMI (Sigma, St. Louis, MO, USA). After adaptation overnight, HGF (25 ng/ml; Sigma) was added to the culture medium and cells were harvested by incubation with 2 mM EDTA (Sigma) for 20 min. Small molecules SU11274 (1.0 µM; Sigma) and PHA665752 (0.1 µM; Calbiochem, Nottingham, UK) dissolved in dimethyl sulfoxide (DMSO) were used for c-MET inhibition.

### Microarray analysis

DU145 cells were stimulated for 2, 8 and 24 hours with HGF or vehicle, and RNA was isolated using RNAzol B reagent (Tel-test Inc., Friendswood, USA). After RNA isolation with chloroform, isopropanol and ethanol, DNA was digested using DNA-free kit (Ambion, Huntingdon, UK). RNA quality and quantity were measured using RNA 6000 Nano kit on a 2100 Bioanalyzer (Agilent, Palo Alto, CA, USA). Samples with RNA integrity numbers of >8.5 were selected. 5 µg of total RNA from stimulated and control samples were used to prepare antisense biotinylated RNA according to manufacturer's one-cycle protocol (Affymetrix, Santa Clara, CA, USA). Hybridization to Affymetrix Human U133plus2.0 GeneChips (54,614 probe sets, representing approximately 47,000 transcripts), staining, washing, and scanning procedures were performed as described by Affymetrix (Affymetrix, Santa Clara, CA, USA), and performed by Erasmus MC Center for Biomics. Microarray data were processed and normalized using the Affymetrix Microarray Suite software. RMA quantile normalization was performed and expression values (EVs) between arrays were normalized by setting the average of each of the 6 arrays to 150; values <30 were set to 30. For each time point ^2^log ratios between HGF and vehicle treated cells were calculated. Array data are MIAME compliant and have been submitted to GEO (GSE16659). Linkage to other databases was performed using SRS7; the Treeview program was used for generating heatmap pictures [23], [24].

### Quantitative real-time PCR

Quantitative real-time PCR was performed using Taqman RXN PCR Core Reagents including MQ, Taq Buffer, MgCl_2_, dNTP, Amplitaq G (Applied Biosystems, Foster City, CA, USA). The following probes were used: Hs02379687_s1 (CD24); Hs00174139_m1 (CD44); Hs01041017_m1 (CD49f); Hs00165814_m1 (SOX9); Hs00158148_m1 (CD49b). The amount of target gene was normalized to GAPDH (Hs99999905_m1).

For analysis of c-MET inhibitors on CD49b, total RNA was isolated with RNAzol B reagent (Tel-Test) according to the manufacturer's protocol. RT reaction was performed with oligo(dT)12–18 primer (Invitrogen, La Jolla, CA). After addition of first-strand buffer, dithithreitol, dNTPs and RNAsin, RT reactions were initiated by MMLV-RT (Invitrogen) and incubated for 1 hour at 37°C. Quantitative PCR was performed using SYBR-green mastermix (Applied Biosystems). CD49b forward 5′-CAG GCA CAC CAA AGA ATT GA-3′, reverse 5′-GAA GAA GCC GAG CTT CCA TA-3′, GAPDH forward 5′-ACT GTG GTC ATG AGT CCT TC-3′, reverse 5′-CAT GTT CGT CAT GGG TG-3′, and PBGD forward 5′-CAT GTC TGG TAA CGG CAA TG-3′ and reverse 5′-GTA CGA GGC TTT CAA TGT TG-3′. Signals were analyzed by ABI Prism 7700 system (Applied Biosystems). Quantity of target genes was determined using standard curves from serial dilutions. For determination of Notch-receptors and ligands, RT-PCR was performed using the primer sets and cycli as depicted in Table S1 at an annealing temperature of 60°C.

### Flow cytometry and Western blotting

For analysis of membranous proteins 0.5×10^6^ DU145 cells were incubated with anti-CD49b (1∶500; Chemicon, Hampshire, UK), anti-CD49f (1∶10,000; Abcam, Cambridge, UK), anti-CD44-FITC (1∶200; BD, Franklin Lakes, NJ, USA), anti-CD24-PE (1∶10; BD) and anti-CD133-PE (clone AC133; 1∶100; Miltenyibiotec; Bergisch Gladbach, Germany), in 50 µl PBS/2% FCS for 30 min. on ice. After washing, cells were incubated with secondary antibodies labelled with Alexa Fluor 488 (1∶500) for 15 min. After suspension in 300 µl PBS/2% FCS with Hoechst 33258 (2 µg/ml) to select for living cells, protein expression was measured using a FACSAria flow cytometer (BD) equipped with three lasers (407, 488 and 633 nm).

For analysis of SOX9 and c-MET protein, stimulated and control cells were lysed in RIPA buffer (10 mM tris pH 7.4, 150 mM NaCl, 1% Triton ×100, 1% deoxycholate, 0.1% SDS, 5 mM EDTA) containing proteinase inhibitors (Complete; Roche, Basel, Switzerland). Protein concentrations were measured using Biorad protein reagents (Biorad Laboratories, München, Germany). 40 µg of total protein was loaded on a 10% SDS-PAGE gel and transferred to nitrocellulose paper (Protan, Schleicher and Schuell, Dassel, Germany). After blocking in 5% NFDM/TBST (Protifar, Nutricia, Zoetermeer, The Netherlands) for 1 hour, blots were incubated at 4°C overnight with 0.2 µg/ml anti-SOX9 (clone AF3075; R&D, Minneapolis, USA) or 1∶1,000 c-MET (clone C12; Santa Cruz). After washing, blots were incubated with 1∶1,000 anti-goat-HRP or anti-rabbit-HRP (Dako, Glostrup, Denmark) for 1 hour, processed using BM Chemiluminescence Blotting Substrate (Roche) and quantified with ImageJ program.

### MTT and cell adhesion assays

5.0×10^3^ DU145 cells were stimulated in 96-wells plates with 25 ng/ml HGF for 0, 3 and 6 days. Cultures were incubated with Thiazolyl blue tetrazolium bromide (MTT 5 mg/ml; Applichem, Darmstadt, Germany) for 4 hours, after which the metabolic product was suspended in 100 µl buffered DMSO and measured at 570 nm with a BIO-RAD 550 microplate reader (Biorad). For quantification of cell adhesion, 3.0×10^5^ stimulated DU145 cells were seeded on 12-wells plates coated with collagen I (Becton Dickinson Labware, Bedford, UK). After adhesion for 15 min., plates were washed, incubated with 1 ml DCC medium containing 5 mg/ml MTT, and optical density was determined.

### Lentiviral infection

HEK 293T cells were seeded in flasks coated with 0.1% gelatin/PBS and grown to 50–60% confluence. Lentiviral particles were produced after transfection of HEK 293T cells with 20 µg of vector DNA (pLKO.1 shRNA, ID167, clone NM_000245.x-502s1c1; Sigma) together with 15 µg pPAX_2_ and 6 µg pMD_2_G using CaPO_4_ precipitation, after which dH_2_O, 2.5M CaCl_2_ and HEPES buffered saline (pH 7.05) was added. The transfection mixture was left to incubate for 20–30 min. at room temperature before it was added to the HEK 293T cells. The shRNA lentivirus-containing cell culture supernatants were collected 24 and 48 hours after transfection, and passed through a 0.45-µm filter. To determine the transfection efficiency HEK 293T cells were simultaneously transfected with Green Fluorescent Protein (GFP). Scrambled shRNA (Sigma) was used as control. DU145 was then infected with the collected virus (1∶1) overnight and infected clones were selected by dilutional cloning.

### Orthotopic injection

1.0×10^5^ DU145 cells were injected in the dorsolateral lobe of male nu/nu Naval Medical Research Institute (NMRI) mice together with 1.0×10^6^ PrSC prostate fibroblasts in 20 µl RPMI/DCC medium containing human HGF (25 ng/ml) [25]. Injections were performed with a 30G microlance needle (Becton Dickinson, Alphen a/d Rijn, The Netherlands) on a Luer tip microliter 700 syringe (Hamilton, Bonaduz, Switserland) after abdominal incision under anesthesia. Tumour volume (TV) was monitored every week by transrectal ultrasonography (Endosonics Europe BV, Rijswijk, The Netherlands). Mice were sacrificed at a TV>1000 mm^3^ or after 3–4 months. The prostate was histologically analyzed together with abdominal lymph nodes and lungs.

### Immunohistochemistry

Immunohistochemistry for c-MET was performed on 94 formalin-fixed, paraffin-embedded radical prostatectomies (RP). Sections of 4 µm were dewaxed and rehydrated using xylene and ethanol. Endogeneous peroxidase was quenched and antigen retrieval was performed during 15 min. of microwave irradiation (700 W) in Tris-EDTA (pH = 9). Slides were incubated with rabbit anti-human c-MET (1∶100; C12; Santa Cruz) overnight at 4°C and visualized using the EnVision system (DAKO). For quantification of c-MET, the presence of strongly positive cells was scored at the perimeter, arbitrarily defined as the outer 2 mm of the tumour and compared with the centre.

For co-localization studies, 6 liquid N_2_ frozen RP slides were acetone-fixed and incubated with anti-c-MET (1∶100), and mouse anti-human CD49b (1∶100; HAS-3; Abcam) or rat anti-human CD49f (1∶100; Abcam) overnight at 4°C. After washing, slides were incubated with swine anti-rabbit biogenin (1∶150; DAKO) combined with anti-mouse or anti-rat antibody labelled with Alexa 488 (1∶100) for 30 min., followed by avidin-Cy3 (1∶100) complex formation for 30 min. at room temperature.

### Statistics

Growth effects of HGF and c-MET inhibitors were evaluated with Student's *t*-test. Immunohistochemical expression of c-MET in RP was analyzed with Pearson χ^2^ test. Tumour formation in mice were evaluated using both tests, all using SPSS version 15.0 (SPSS Inc, IL, USA). A two-sided p-value below 0.05 was considered significant.

## Results

### Discovery of HGF/c-MET regulated genes

**Figure 1:**
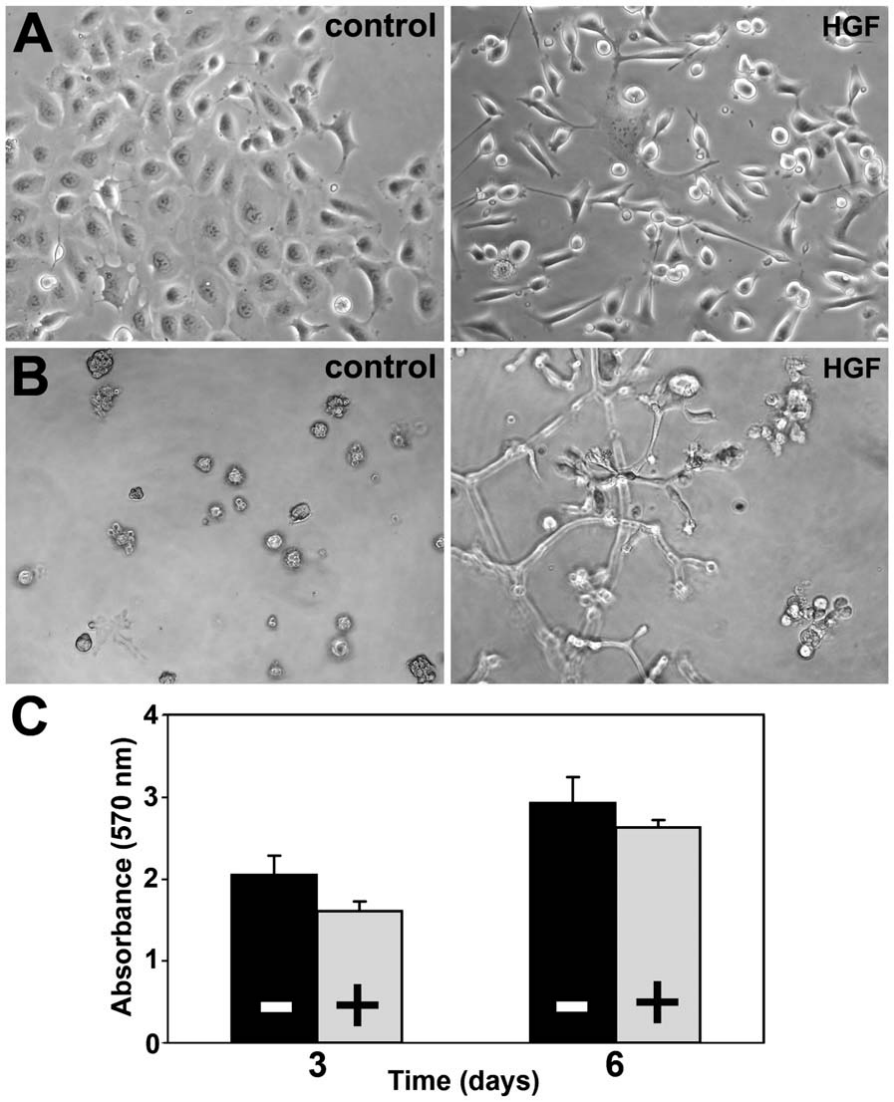
Effects of HGF on DU145 cell line. *A*, HGF induced transformation from epithelioid cell clusters towards single spindle cells in 2-dimensional culture. *B*, In 3-dimensional Matrigel sprouts originated from compact cellular nodules after HGF stimulation. *C*, Cell proliferation is inhibited by 21% (p<0.003) after 3 days and 10% (p = 0.024) after 6 days of HGF stimulation (control *black bars* −; HGF *grey bars* +). *A*, *B* Original magnification 40×.

Previous RT-PCR and Northern blot analysis demonstrated that c-MET is expressed in androgen-independent prostate cancer cell lines DU145 and PC3, but not in androgen-dependent LNCaP [18], [20]. Stimulation of DU145 with HGF resulted in cell scattering and migration in 2D-culture (Figure 1A), while stellate sprouts form in 3D Matrigel matrices (Figure 1B). During scattering, DU145 cells obtained a spindle form in contrast to their normal epithelioid shape, while cell growth was significantly inhibited by 21% after 3 days (p<0.003) and 10% after 6 days (p = 0.024)(Figure 1C). Although PC3 possesses c-MET protein, HGF stimulation did not induce morphological changes (unpublished observations) putatively due to its lack of a functional α-catenin/E-cadherin complex [26].

**Figure 2:**
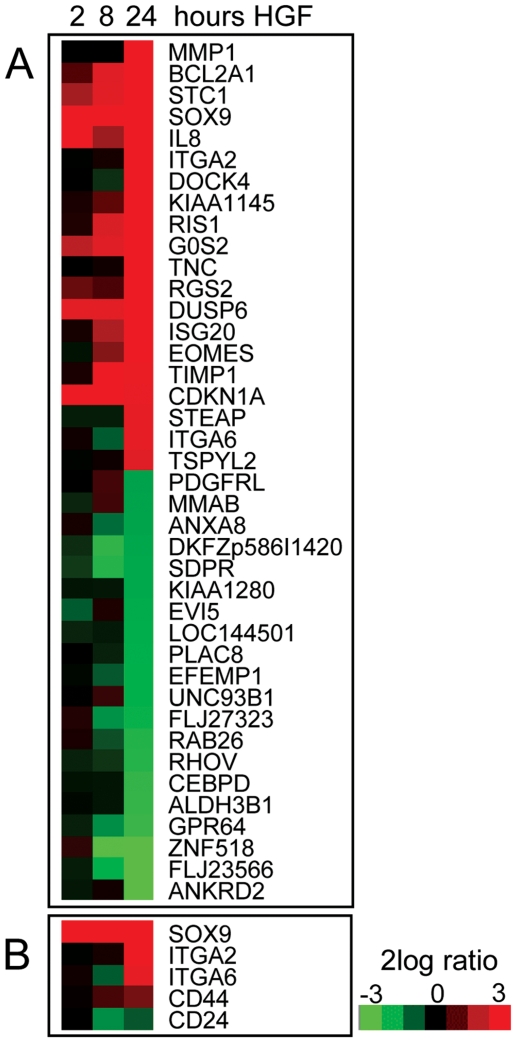
Effects of HGF on gene-expression of DU145 cell line. *A*, Top 20 up- and down-regulated genes after HGF stimulation for 24 hours, combined with respective gene-expression profiles after stimulation for 2 and 8 hours. *B*, Effect of HGF on genes associated with prostate stem cell phenotype.

To explore the molecular pathways relevant for c-MET function in prostate cancer, we performed microarray expression analysis of DU145 cells stimulated with HGF. Genes were selected based on a two-fold up or down-regulation by at least one probe set at the 24 hours' time point and an average expression with a 1.41 fold difference. In total, 371 genes fulfilled these criteria of which 238 were up- and 133 down-regulated by HGF (Table S2); the top 20 up- and down-regulated genes are depicted in Figure 2A.

We found that various membranous markers utilized for stem cell selection were enhanced after 24 hours: α_2_-integrin/CD49b, α_6_-integrin/CD49f and CD44, while CD24 was down regulated (Figure 2B). The transcription factor SOX9 was also induced by HGF. SOX9 is required for early differentiation of prostate epithelium during embryogenesis and is reactivated in prostate carcinogenesis implicating a relevant function in immature prostate cells [27]. Expression of two other putative prostatic stem cell markers CD133 and LGR5 was below detection limits [14], [28].

### Validation of phenotypic stem-like cell induction

**Figure 3:**
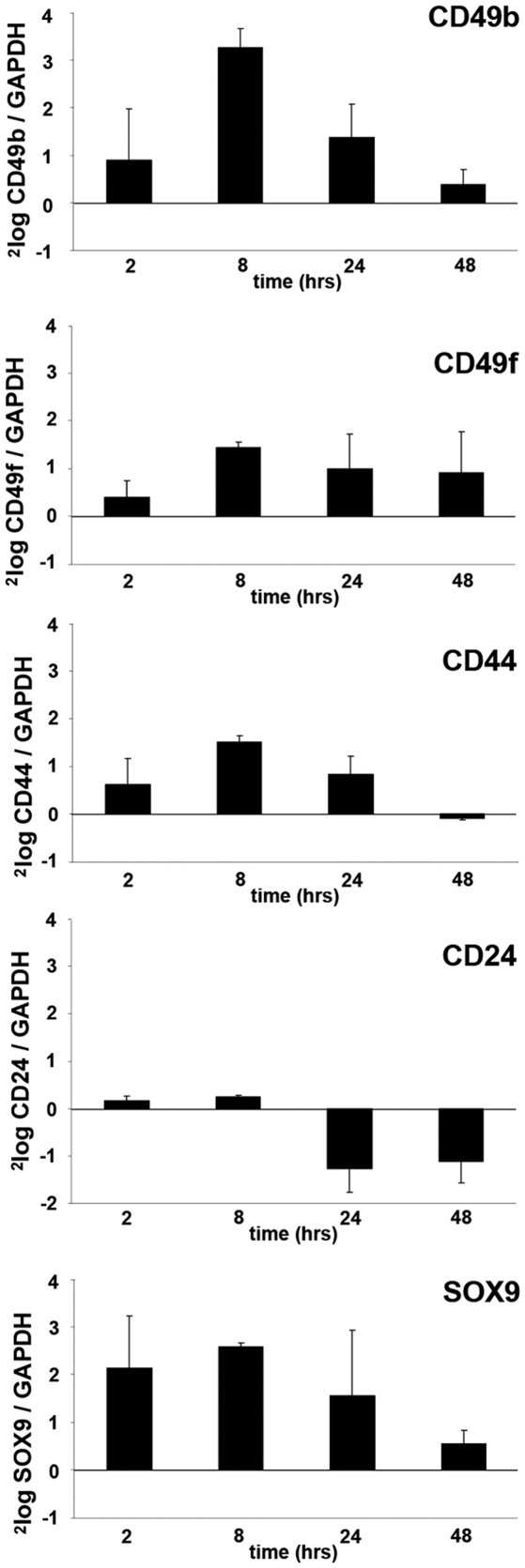
Validation of stem cell associated gene-expression after HGF stimulation. Quantitative real-time PCR confirmed induction of CD49b, CD49f, CD44 and SOX9 mRNA, together with down-regulation of CD24.

**Figure 4:**
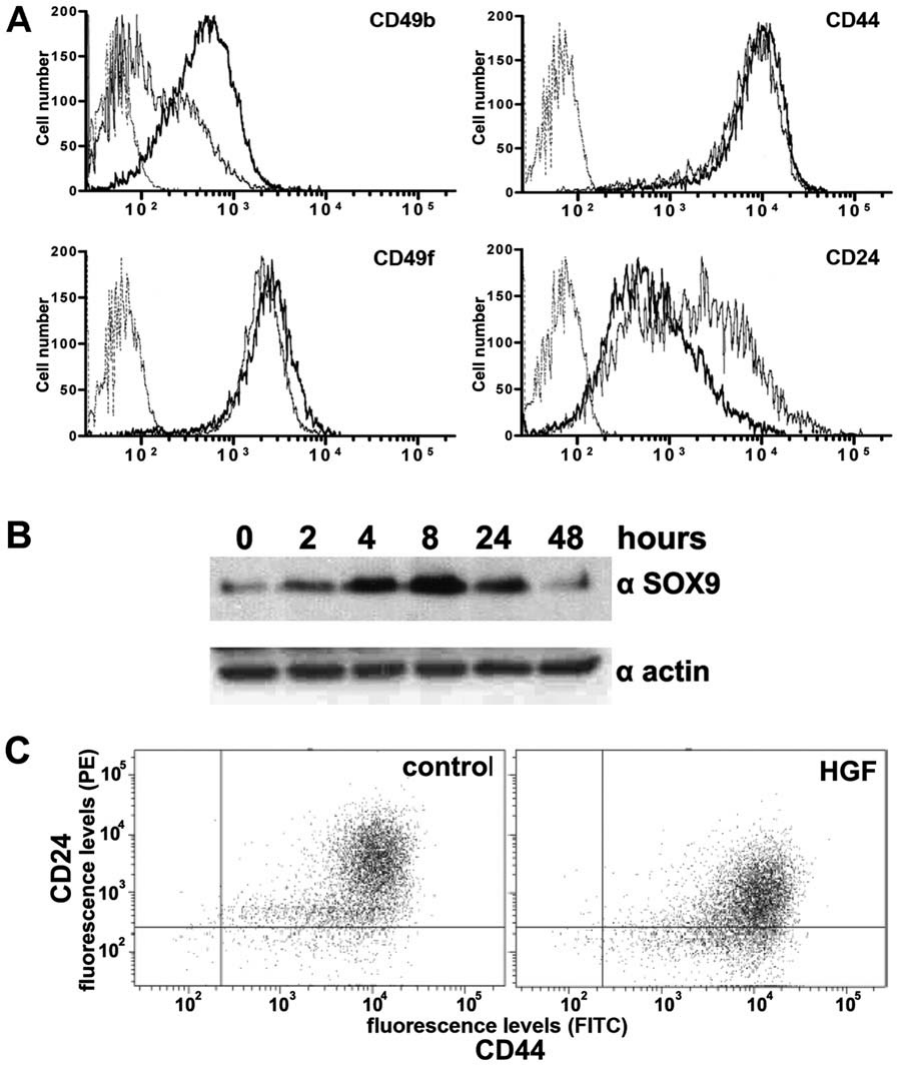
Validation of stem cell associated proteins after HGF stimulation. *A*, FACS demonstrated up-regulation of CD49b (240%), CD49f (22%), CD44 (26%), together with down-regulation of CD24 (−69%) after HGF stimulation. Thin gray line represents labelling with secondary antibody only (negative control), thin black line DU145 without HGF, and thick black line DU145 with HGF. *B*, Western blot showed enhancement of SOX9 expression upon HGF stimulation from 2 to 24 hours. *C*, FACS demonstrated induction of double-labelled CD44^+^/CD24^−^ DU145 cells (control 8% *versus* HGF 26%).

Regulation of stem cell associated genes by the HGF/c-MET pathway was validated in three independent DU145 experiments using quantitative PCR. A regulatory effect of HGF was demonstrated for all selected genes (Figure 3). After 24 hours of stimulation CD24 (0.4 fold) was down-regulated, while CD49b (2.6 fold), CD49f (2.0 fold), CD44 (1.8 fold) and SOX9 (2.9 fold) were all enhanced. Subsequently, FACS analysis confirmed up-regulation of membranous proteins CD49b, CD49f, CD44, and suppression of CD24 (Figure 4A). The effects on protein expression were most prominent for CD49b with a general increase of 240%. Membranous expression of CD49f and CD44 was only slightly enhanced after HGF stimulation (22% and 26%, resp.). Since SOX9 is localized in the nucleus, we performed Western blot analysis to confirm its regulation by HGF. As expected, expression of SOX9 protein was strongly induced in stimulated DU145 cells up to 521% after 24 hours (Figure 4B).

Both CD44^+^/CD24^−^ and α_2_β_1_-integrin^+^/CD133^+^ profiles select for stem-like cells in prostate cancer [4], [14]. Upon HGF stimulation, the CD44^+^/CD24^−^ population was enriched 3.25 fold (from 8% to 26%) combined with a 0.8 fold decrease (from 91% to 73%) of more mature CD44^+^/CD24^+^ cells (Figure 4C). Selection for α_2_β_1_-integrin^+^/CD133^+^ cells is often pursued at first by short-term adhesion to collagen I matrix, which selects for high-integrin expressing cells [3]. To verify whether c-MET activation enhances the fraction of rapidly attaching immature cells, we quantified adhesion to collagen I after 15 min. Within this period, 0.037% (mean; SD 0.006%) of control cells attached to collagen I. HGF activation of DU145 cells significantly (p<0.01) enriched for rapidly adhering cells (mean 0.055%; SD 0.011%). With FACS analysis, CD133 expression in DU145 was below detection limits and not affected by HGF stimulation (data not shown).

### HGF activates Notch pathway

**Figure 5:**
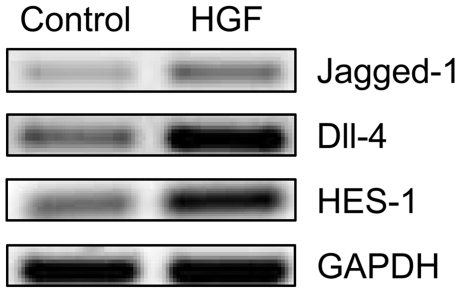
Effects of HGF on Notch pathway in DU145 cell line. HGF stimulation led to over-expression of Notch ligands Jagged-1 and Dll-4 RNA, and the down-stream Notch target HES-1.

Notch signalling plays an important role in the prostate development and has been implicated in CSC function [29]–[31]. Analysis of the gene-expression profile demonstrated over-expression (3.0×) of the down-stream Notch target HES-1 upon HGF stimulation. Therefore, we investigated the effects of HGF on the Notch receptors and their ligands. We found that c-MET activation led to up-regulation of Notch ligands Jagged-1 and Delta-like (Dll) 4 (Figure 5). The ligand Jagged-2, receptors Notch-2 and Notch-3 were unaffected, while Notch-1 receptor was down-regulated upon HGF stimulation (data not shown), implicating that Notch activation resulted from over-expression of ligands Jagged-1 and Dll-4.

#### Inhibitors of c-MET block development of stem-like phenotype

**Figure 6:**
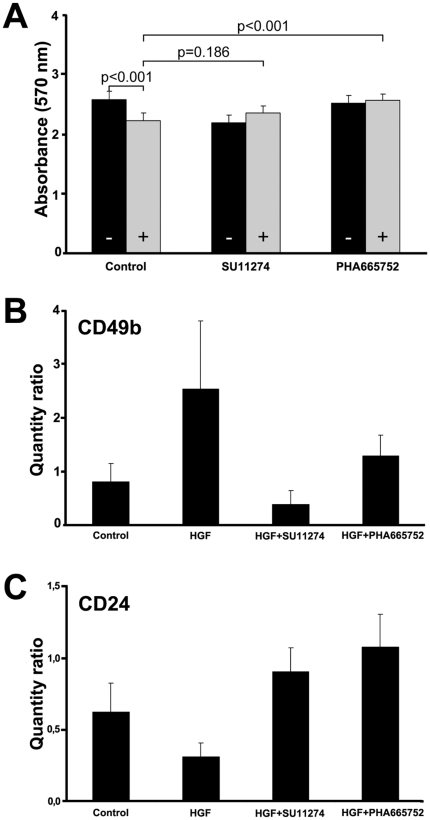
Effects of c-MET inhibitors on DU145 cell line. *A*, Cell proliferation is inhibited by SU11274 in both control (*black bars* −) and HGF stimulated cells (*grey bars* +), so that it did not reverse HGF mediated growth reduction (p = 0.186). PHA665752 did not affect cell proliferation in control cells, and blocked HGF induced growth inhibition (p<0.001). *B*, *C*, Both SU11274 and PHA665752 blocked HGF mediated induction of CD49b (*B*) and inhibition of CD24 (*C*). Standard deviations represent three independent experiments.

Recognition of immature cells in human malignancies initiated development of specific pharmaceutical agents targeting this biologically important population. The small molecules SU11274 and PHA665752, which both inhibit c-MET enzymatic activity completely blocked cell scattering in culture and formation of stellate sprouts in Matrigel (data not shown) [32], [33]. HGF-mediated growth reduction was significantly reverted to the baseline level (p<0.001) by PHA665752, but not by SU11274 (p = 0.186)(Figure 6A). SU11274 (p<0.001) and PHA665752 (p = 0.016) significantly reversed both the induction of CD49b (Figure 6B) as well as the inhibition of CD24 (both p<0.001) within 24 hours (Figure 6C). Because HGF only moderately affected CD49f and CD44 levels, we did not evaluate pharmacological effects on these markers.

### Expression of c-MET modulates efficient tumour-formation *in vivo*

**Figure 7:**
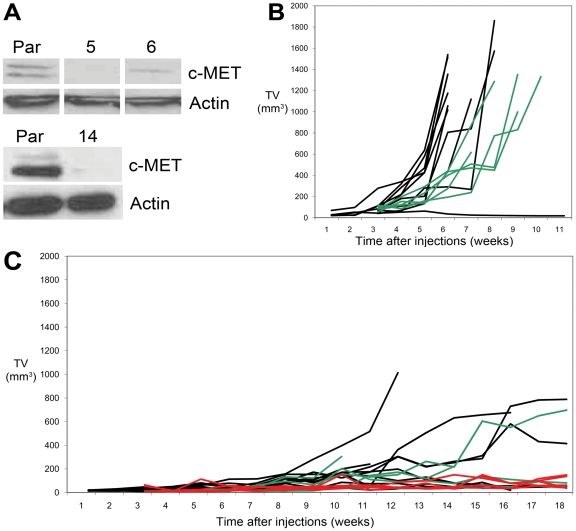
Reduced orthotopic tumour-formation of DU145 in NMRI mice after knock-down of c-MET. *A*, Three DU145 clones (5, 6, 14) with low to negative c-MET protein expression (Western blot) were generated by stable shRNA infection. *B*, Orthotopic injection of 1.0×10^5^ DU145 with functional c-MET led to efficient tumour-formation in 9/10 parental (*black*) and 5/5 control DU145 cells (*green*) infected with scrambled RNA. *C*, Orthotopic tumour-formation was significantly reduced in DU145 with low c-MET expression. A total of 6/10 DU145Sh167#14 (*black*), 3/5 DU145Sh167#5 (*green*) and 2/5 DU145Sh167#6 (*red*) injections resulted in tumour-formation. Only one tumour reached a TV of 1000 mm^3^ during the study period of 52–124 days.

For analyzing the role of c-MET in tumour-formation *in vivo*, we infected DU145 cells with lentivirus expressing shRNA targeting c-MET receptor and selected three c-MET negative clones (Figure 7A). The c-MET negative DU145 clones had similar morphology as the parental line, when grown in 2D or 3D culture, but did not react to HGF by scattering or formation of stellate sprouts (data not shown). Orthotopic injection of parental DU145 resulted in 90% (9/10) tumour formation; these mice were sacrificed with a TV of 1357 mm^3^ (mean; range 1016–1860 mm^3^) after 42.6 days (mean; range 38–52 days). Control DU145 cells infected with scrambled shRNA led to tumour formation in 100% (5/5). These mice were all sacrificed due to large TV after 59 days (mean; range 52–66 days)(Figure 7B). Significant reduction of tumour formation occurred in the DU145 clones with silenced c-MET. In total 55% (11/20) of the clones (6/10 Sh167#14, 3/5 Sh167#5 and 2/5 Sh167#6) gave rise to tumours, of which only one was sacrificed due to large TV (1013 mm^3^; 81 days). Fifteen mice were sacrificed at 119 days (mean; range 109–124 days), of which 6 had small tumours (mean TV 477 mm^3^; range 137–788 mm^3^). Four mice died untimely between 52 to 102 days, all showing small tumours (TV mean 225 mm^3^; range 137–304 mm^3^)(Figure 7C). In none of the mice metastases were observed. Taken together, functional c-MET mediated efficient tumour formation in parental DU145 (p<0.02) and resulted in significantly larger tumours in control DU145 cells (p<0.001).

#### Cells expressing c-MET are located at invasive front of prostate cancer

**Figure 8:**
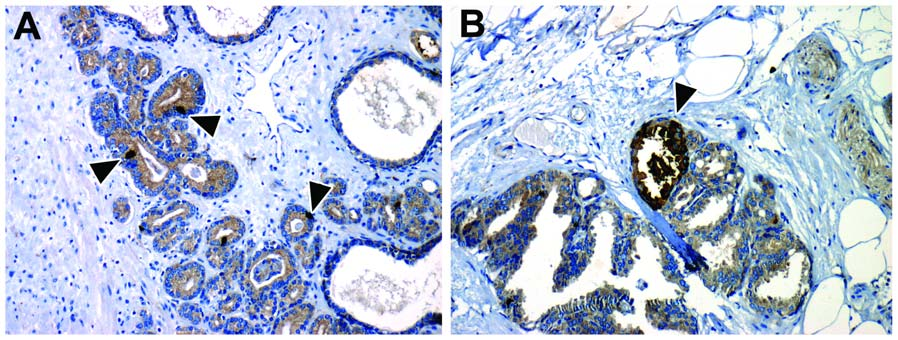
Immunohistochemical expression of c-MET in human prostate cancer. c-MET is highly expressed in scattered prostate cancer cells (*A*), and particularly at invasive fronts within peri-prostatic fat tissue (*B*); arrowheads indicate positive cells. Original magnification 100×.

Tumour cells with high c-MET expression are specifically enriched at the invasive front of colorectal carcinomas, where they mediate EMT, migration and matrix invasion [34], [35]. To determine c-MET expression at the invasive front in prostate cancer, we compared the presence of high c-MET expressing cells at the perimeter and the centre of well-fixed RP specimens (N = 94). Overall high c-MET expressing cells (Figure 8A) were found in 37 cases (39.4%). The perimeter contained more high c-MET expressing cells than the tumour centre in 27 cases (73.0%). In 6 cases (16.2%) these cells were more abundant in the centre, while in 4 RP's (10.8%) there was no difference in expression (Pearson χ^2^; p<0.001). Expression of c-MET in pre-existent benign basal cells within the entire slide served as an internal control to exclude fixation-induced staining artefacts. In total 25 cases revealed tumour expansion into extra-prostatic fat tissue; in 10 extra-prostatic tumour areas (40.0%) high c-MET expressing cells were encountered (Figure 8B).

**Figure 9:**
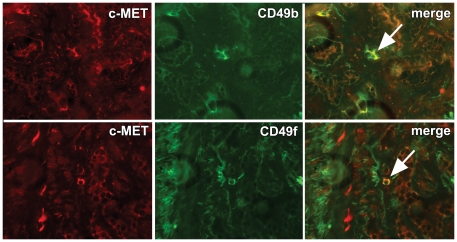
Validation of c-MET co-expression with stem-like cell markers in human prostate cancer. Immunofluorescent double-labelling of c-MET (Cy3; red) with CD49b or CD49f (Alexa 488; green). Co-expression of c-MET with both CD49b and CD49f was present in scattered prostate cancer cells (*arrows*). Original magnification 100×.

Finally, co-expression studies were performed in RP specimens to validate c-MET co-expression with stem-like cell markers in patients. Immunofluorescent stainings for c-MET, CD49b and CD49f demonstrated diffuse expression of all markers in basal cells of pre-existent glands. Expression was low to absent in the vast majority of normal and malignant luminal cells. Receptor c-MET was expressed sporadically at high levels in single malignant cells. These cells co-expressed CD49b and CD49f, indicating that c-MET positive cells indeed co-express a stem-like cell phenotype in human prostate cancer (Figure 9).

## Discussion

At least three different cell populations can be discriminated in human prostate cancer. While differentiated exocrine and neuro-endocrine cells represent the predominant cell types, evidence is increasing that a minor population of immature cells is determinative for the biological behaviour of prostate cancer. Analogical to normal glandular prostate epithelium, Collins et al. distinguished immature stem (α_2_β_1_-integrin^+^/CD133^+^) and transit-amplifying cells (α_2_β_1_-integrin^+^/CD133^−^) in human prostate cancer specimens by clonogenic, proliferative and differentiating capacity *in vitro* [14]. Although CD133 is proposed as a general marker for tumour progenitor cells, its specificity for actual tumour stem cells is still under debate [10]–[12], [14], [36], [37]. In colon cancer, for instance, it has been proposed that the CD44^+^/CD24^−^/CD133^−^ profile represents the tumour stem cell population while CD133^+^ rather marks more differentiated cells [38]. Likewise, in prostate cancer no agreement exists on whether CD133 enriches for stem-like cells [8], [39].

The HGF/c-MET pathway might play a key role in prostate carcinogenesis and tumour progression [18], [19]. In this study, we found HGF to induce a phenotypic profile, which is strongly associated with stem-like cells, namely up-regulation of CD44, CD49b, SOX9 and CD49f, and down-regulation of CD24 on RNA and protein levels. Further, c-MET activation led to a 3.25-fold enrichment of CD44^+^/CD24^−^ cells, representing more accurately the stem-like cell profile for breast and potentially prostate cancer [4], [13]. Activation of c-MET also activated the Notch-signalling pathway by up-regulation of its ligands Jagged-1 and Dll-4, as well as the transcription factor SOX9, which are all important for embryonic prostate development and have been linked to malignant stem-like cells in several organs [27], [31], [40], [41].

A limitation for investigating the HGF/c-MET pathway in human prostate epithelium is the availability of relevant models. Neither prostate cancer cell lines LNCaP, DuCap, VCap, PC346C, 22RV1 nor primary benign epithelial cultures express the c-MET receptor (data not shown). Furthermore, the human c-MET receptor in prostate cancer xenografts has low sensitivity for murine HGF, so that external human HGF sources or transgenic mice are required for functional HGF/c-MET analysis [42]. The only other c-MET positive cell line PC3 shows a stellate growth pattern in 3D Matrigel and scattering in 2D culture, even without stimulation of HGF. Therefore, its growth pattern without HGF resembles the morphogenesis of DU145 with HGF stimulation. We hypothesize that the specific PC3 growth pattern results from a deficient E-cadherin complex function [26]. Interestingly, HGF/c-MET can interfere with E-cadherin function in several ways: a) up-regulation of Snail, Twist and Slug inhibit E-cadherin transcription, b) tyrosine phosporylation of β-catenin leads to a deficient membranous E-cadherin complex, and c) E-cadherin can be cleaved extracellularly by matrilysin [43]–[46]. A functional E-cadherin pathway therefore seems a prerequisite for HGF-induced morphogenesis.

Considering the lack of alternative HGF/c-MET models for prostate cancer, we specifically aimed at validating our results in human prostate cancer specimens. While various genes have been implicated in cancer stemness using *in vitro* and mice models, investigation of respective markers is relatively rarely performed in human specimens. Such validation might disclose discordances in case a hypothesized stem cell marker is expressed in the vast majority of cancer cells (for instance BMI-1), or is not expressed in respective cancer tissue at all [47]. Previously, we have demonstrated that c-MET is over-expressed in scattered prostate cancer cells at radical prostatectomies [18]. In this study, we demonstrate that high c-MET expressing cells preferentially occur at the periphery of prostate cancer and in areas of extra-prostatic extension. This is in line with the preferential presence of stem-like cells at the invasive front of colorectal cancer, as identified by their nuclear expression of beta-catenin [34]. By immunofluorescent double-labelling we were able to confirm that c-MET positive cells co-expressed stem-like markers CD49b and CD49f in surgical specimens. Despite the limited availability of c-MET models in prostate cancer, these results indicate that HGF-mediated induction of a stem-like phenotype is representative for human disease.

The induction of a stem-like phenotype has recently also been demonstrated in two other models. In immortalized human mammary epithelial cells ectopic expression of Snail or Twist transcription factors, which both inhibit E-cadherin expression, led to EMT and induction of the CD44^+/^CD24^−^ stem cell phenotype [15]. After EMT, the cell line exhibited properties of stem cells by efficient formation of mammaspheres, differentiation towards myo-epthelial and luminal cells, and high tumour-forming capacity after malignant transformation with tumourigenic V12H-ras oncogene. In colon cancer, myofibroblast-secreted HGF restored a stem-like phenotype in more differentiated cells *in vitro* and *in vivo* [16]. Our data are in line with these results, demonstrating that stimulation of more mature prostate cancer cells with HGF can induce a stem-like marker profile and high tumour-formation *in vivo*. Interestingly, these observations support a close link between stem-like cells and tumour infiltration. While it is well known that c-MET activation leads to enhanced invasive capacity together with secretion of matrix degrading proteins such as uPA, MMP-1 and MMP-9, we now demonstrate that a stem-like phenotype is acquired simultaneously [18], [48], [49]. Klarmann et al. also found such a relation as LNCaP cells invasive in Matrigel obtained stem-like characteristics, while Collins et al. demonstrate that α_2_β_1_-integrin^+^/CD133^+^ cells isolated from RP specimens have high invasive capacity [14], [50]. Taken together, these results suggest that stem-like cells represent a temporary state-of-being during tumour invasion. If appropriate stimulation such as HGF is discontinued, cellular invasion might subsequently be terminated, after which stem-like cells undergo differentiation.

In conclusion, activation of the HGF/c-MET pathway gives rise to a stem-like phenotype, preferentially at the invasive front of human prostate cancer. Stem-like cells therefore might represent a dynamic and inducible population, mediating invasion at the perimeter of prostate cancer. Inhibition of c-MET has potency in blocking stem-like cell transition and therefore is a promising tool for targeted therapy of prostate cancer.

## Supporting Information

Table S1 **Primers and cylci number for RT-PCR of Notch receptors and ligands.**
(PDF)

Table S2 **HGF induced gene-expression in DU145 cell line.**
(PDF)
