# Supplementary material for: Activation of c-MET Induces a Stem-Like Phenotype in Human Prostate Cancer
Source: PLoS One. 2011 Nov 14;6(11):e26753. doi: 10.1371/journal.pone.0026753 (PMC3215704; doi:10.1371/journal.pone.0026753)
Supplement: Table S1 — Primers and cylci number for RT-PCR of Notch receptors and ligands. (PDF) [file pone.0026753.s001.pdf]

**Table S1. Primers and cylci number for RT-PCR of Notch receptors and ligands**

| Gene | Cylci | Forward primer<br><br>5' – 3' | Reverse primers<br><br>5' – 3' |
|---|---|---|---|
| Dll-1 | 29 | AGGACCTCAAGGGTGACGAC | GAAGTTGAACAGCCCGAGTC |
| Dll-4 | 29 | GAAAAGCCAGAGTGTCGGATA | AATGCAGAAGGAAGGTCCAG |
| HES-1 | 29 | CGGACATTCTGGAAATGACA | GTGCGCACCTCGGTATTAAC |
| Jagged-1 | 29 | AAACGTGATGGAAACAGCTC | AGTAGAAGGCCGTCACCAAG |
| Jagged-2 | 24 | GGAACAGCTCACTGCTCCTG | CTCCTCTCCCGCTCTTTCCT |
| Notch-1 | 28 | GCAGTTGTGCTCCTGAAGAA | CGGTCCATATGATCCGTGA |
| Notch-2 | 28 | GGATGTGAATGCAGTGGATG | CAGGATCTTGGCTGCTTCAT |
| Notch-3 | 28 | GGATGAGCTTGGGAAATCAG | CAGCTTGGCAGCCTCATAG |
