# Supplementary material for: Activation of c-MET Induces a Stem-Like Phenotype in Human Prostate Cancer
Source: PLoS One. 2011 Nov 14;6(11):e26753. doi: 10.1371/journal.pone.0026753 (PMC3215704; doi:10.1371/journal.pone.0026753)
Supplement: Table S2 — HGF induced gene-expression in DU145 cell line. (PDF) [file pone.0026753.s002.pdf]

Table S2. HGF induced gene-expression in DU145 cell line

Gene Identifiers			2log ratios HGF *versus* no HGF			overall raw expression
UniGene	Gene symbol	LocusLink	2 hours	8 hours	24 hours	EV
Hs.83169	MMP1	4312	0,04	0,04	4,75	265,2
Hs.227817	BCL2A1	597	0,73	1,78	3,51	124,1
Hs.25590	STC1	6781	1,33	1,80	3,16	191,1
Hs.2316	SOX9	6662	1,88	2,46	2,53	249,5
Hs.624	IL8	3576	2,21	1,27	2,49	873,7
Hs.482077	ITGA2	3673	-0,07	0,24	2,32	139,7
Hs.118140	DOCK4	9732	-0,02	-0,41	2,24	101,3
Hs.370410	KIAA1145	57458	0,26	0,81	2,23	37,5
Hs.35861	RIS1	25907	0,33	1,74	2,16	638,2
Hs.432132	G0S2	50486	1,50	1,78	1,94	882,5
Hs.143250	TNC	3371	0,04	0,19	1,93	40,6
Hs.78944	RGS2	5997	0,88	0,68	1,92	673,8
Hs.298654	DUSP6	1848	1,81	1,81	1,91	345,0
Hs.459265	ISG20	3669	0,25	1,40	1,91	264,2
Hs.147279	EOMES	8320	-0,17	1,08	1,89	80,4
Hs.522632	TIMP1	7076	0,26	2,36	1,86	2858,1
Hs.370771	CDKN1A	1026	2,14	2,51	1,84	374,0
Hs.61635	STEAP	26872	-0,26	-0,26	1,83	230,4
Hs.133397	ITGA6	3655	0,19	-0,75	1,83	118,6
Hs.136164	TSPYL2	64061	-0,08	0,18	1,78	178,3
Hs.306322	NAV3	89795	-0,56	0,14	1,72	29,8
Hs.10082	KCNN4	3783	0,31	1,59	1,71	176,3
Hs.24907	CORO2B	10391	0,00	1,42	1,67	16,9
Hs.323308	SPRY4	81848	1,20	1,57	1,63	44,5
Hs.106857	CALB2	794	-0,16	0,37	1,60	54,5
Hs.406691	HIST1H2AJ	8331	-0,25	-1,03	1,59	80,4
Hs.326035	EGR1	1958	1,73	2,24	1,59	884,8
Hs.250666	HES1	3280	0,92	1,08	1,58	68,5
Hs.459709	PRSS22	64063	0,51	1,27	1,57	74,8
Hs.516484	S100A2	6273	0,32	1,63	1,57	565,2
Hs.103527	SH2D2A	9047	0,00	1,06	1,56	46,2
Hs.486818	TTF2	8458	0,35	-0,18	1,53	101,5
Hs.789	CXCL1	2919	1,99	0,84	1,52	256,8

Gene Identifiers			2log ratios HGF *versus* no HGF			overall raw expression
UniGene	Gene symbol	LocusLink	2 hours	8 hours	24 hours	EV
Hs.154299	F2RL1	2150	0,43	0,55	1,52	118,4
Hs.371240	AKAP12	9590	0,30	0,51	1,51	165,7
Hs.89690	CXCL3	2921	1,21	0,19	1,50	54,4
Hs.157726	AK000477	440462	0,35	0,36	1,49	25,8
Hs.411311	IL24	11009	0,05	0,97	1,49	45,0
Hs.443727	LHX1	3975	1,24	0,91	1,49	73,5
Hs.472054	C20orf42	55612	0,09	0,18	1,45	105,7
Hs.9613	ANGPTL4	51129	1,89	2,34	1,44	299,8
Hs.79741	FLJ10116	55686	-0,08	0,48	1,44	137,7
Hs.127189	CA13	377677	0,40	0,19	1,42	116,6
Hs.2030	THBD	7056	0,00	1,25	1,41	43,0
Hs.466871	PLAUR	5329	0,81	1,80	1,41	1176,1
Hs.268887	STK17A	9263	0,17	0,33	1,41	232,9
Hs.2399	MMP14	4323	-0,43	0,58	1,40	39,9
Hs.345139	GEM	2669	0,46	0,62	1,40	94,7
Hs.503763	LOC91801	91801	-0,33	-0,56	1,40	98,4
Hs.506784	LNK	10019	0,22	1,30	1,40	142,5
Hs.521540	LETM2	137994	0,00	0,99	1,40	58,1
Hs.524528	CYP27B1	1594	0,21	0,77	1,39	131,0
Hs.155342	PRKCD	5580	-0,01	1,45	1,38	198,1
Hs.464848	B4GALT6	9331	0,08	-0,55	1,37	59,9
Hs.8261	SSB1	80176	0,12	1,24	1,36	36,5
Hs.133539	KIAA0303	23227	0,30	0,79	1,35	42,1
Hs.149156	GLDC	2731	0,07	0,79	1,34	225,9
Hs.444959	ACOX2	8309	-0,31	0,96	1,34	186,5
Hs.46523	ELK3	2004	-0,01	0,05	1,34	432,1
Hs.99196	MGC11324	84803	0,28	0,14	1,33	346,3
Hs.107740	KLF2	10365	0,63	1,38	1,33	49,6
Hs.368077	SERPINB8	5271	0,42	0,46	1,33	65,7
Hs.505654	ITGA5	3678	0,01	1,36	1,32	280,6
Hs.517400	PEX26	55670	-0,08	0,31	1,32	87,6
Hs.799	DTR	1839	1,51	1,33	1,31	283,3
Hs.3843	DUSP7	1849	0,20	1,86	1,31	87,1
Hs.68061	SPHK1	8877	0,25	1,82	1,31	86,3
Hs.481720	MYO10	4651	-0,14	0,01	1,28	55,8

Gene Identifiers			2log ratios HGF *versus* no HGF			overall raw expression
UniGene	Gene symbol	LocusLink	2 hours	8 hours	24 hours	EV
Hs.489365	AP1S1	1174	0,25	-0,69	1,28	217,1
Hs.247828	RPL23AP7	118433	-0,36	0,05	1,27	139,8
Hs.435003	RCE1	9986	0,10	1,06	1,27	62,8
Hs.283378	LOC253981	253981	-0,07	0,00	1,26	338,5
Hs.435120	KIF1C	10749	-0,45	1,04	1,25	31,7
Hs.438231	TFPI2	7980	0,91	0,14	1,25	2180,3
Hs.166313	RGS17	26575	0,20	-0,31	1,24	46,3
Hs.546297	SLC20A1	6574	0,46	1,06	1,24	1456,4
Hs.170473	PLEK2	26499	0,15	1,34	1,23	450,8
Hs.111164	MGC33993	221687	-0,25	0,84	1,22	93,9
Hs.149098	SMTN	6525	0,30	1,74	1,22	40,5
Hs.468099	MGC11061	84272	-0,01	-0,76	1,22	48,7
Hs.505777	DDIT3	1649	1,24	-0,06	1,22	243,3
Hs.440025	KIAA0247	9766	0,47	0,19	1,21	101,9
Hs.101150	KIAA1949	170954	0,25	2,10	1,20	868,9
Hs.162902	AOF1	221656	0,19	-0,50	1,20	94,3
Hs.391828	PARD6B	84612	0,77	-0,41	1,20	162,4
Hs.418123	CTSL	1514	0,51	0,94	1,20	660,7
Hs.379821	BJ-TSA-9	84985	0,17	1,77	1,20	543,9
Hs.250072	SLC4A7	9497	0,33	-0,42	1,19	156,7
Hs.507162	FLJ12750	79720	1,09	1,68	1,19	126,3
Hs.476093	CDCP1	64866	0,48	0,95	1,19	720,1
Hs.504187	ASAM	79827	0,09	-0,03	1,18	31,5
Hs.513971	FLJ34018		0,19	-1,00	1,18	79,7
Hs.519313	AF5Q31	27125	0,07	-0,53	1,17	58,0
Hs.417962	DUSP4	1846	1,01	0,50	1,16	77,5
Hs.503165	CENTD2	116985	0,02	1,21	1,16	82,0
Hs.271272	BTBD11	121551	-0,08	1,27	1,16	45,4
Hs.512776	LTBP2	4053	-0,16	-0,02	1,16	55,3
Hs.518805	HMGA1	3159	0,17	1,12	1,16	1022,4
Hs.480694	KIAA1223	57182	0,46	-0,43	1,15	92,4
Hs.149239	EFNB2	1948	0,32	0,49	1,15	32,0
Hs.189075	PTK9	5756	0,05	-0,99	1,15	280,6
Hs.521989	PDCD1LG1	29126	1,36	0,65	1,15	66,4
Hs.187459	ZDHHC14	79683	0,48	0,15	1,14	25,7

Gene Identifiers			2log ratios HGF *versus* no HGF			overall raw expression
UniGene	Gene symbol	LocusLink	2 hours	8 hours	24 hours	EV
Hs.431550	MAP4K4	9448	-0,10	-0,90	1,14	68,0
Hs.4892	Clone 24841		0,00	0,04	1,13	44,2
Hs.97316	NGEF	25791	0,18	1,35	1,13	26,8
Hs.111554	ARL7	10123	0,19	1,01	1,13	76,6
Hs.224607	SDC1	6382	0,27	1,16	1,13	862,9
Hs.505516	KIAA1463	57609	0,16	-0,03	1,13	37,4
Hs.104879	SERPINB9	5272	0,21	0,25	1,12	84,9
Hs.466937	PPP1R13L	10848	-0,24	0,92	1,12	74,9
Hs.517228	TIAM1	7074	0,14	0,09	1,12	86,4
Hs.162963	ANTXR2	118429	0,48	0,34	1,12	49,4
Hs.283148	RAB38	23682	-0,47	0,26	1,11	56,2
Hs.446705	RFFL	117584	-0,01	0,45	1,11	39,4
Hs.471768	MGC4796	83931	1,19	1,75	1,11	44,7
Hs.529322	APG16L	55054	1,04	1,14	1,11	51,4
Hs.202517	DKFZp762O076	55529	-0,30	0,67	1,10	71,9
Hs.465529	MIDN	90007	1,99	0,13	1,10	59,2
Hs.530509	LAMC2	3918	0,09	0,05	1,10	154,4
Hs.40910	CPNE8	144402	-0,01	-0,33	1,10	137,4
Hs.23862	PHCA	55331	-0,19	-1,04	1,10	162,4
Hs.8379	CS0DJ001YJ05		0,72	-0,31	1,09	192,6
Hs.189641	SEC24D	9871	0,22	0,67	1,09	31,6
Hs.526928	SLC35E4	339665	0,00	1,76	1,09	49,6
Hs.98445	FLJ21652		0,31	-0,73	1,08	353,0
Hs.199248	PTGER4	5734	0,93	0,36	1,08	210,9
Hs.436298	EMP1	2012	0,45	0,59	1,08	43,8
Hs.486596	NHSL1	57224	0,76	0,69	1,08	24,0
Hs.531406	LOC286073	286073	-0,19	-0,22	1,08	110,5
Hs.28988	GLRX	2745	-0,18	-0,27	1,08	196,6
Hs.444947	TRIB1	10221	2,00	1,78	1,07	182,8
Hs.477921	WWTR1	25937	0,19	0,38	1,07	505,7
Hs.505924	HMGA2	8091	0,00	0,10	1,07	15,3
Hs.488240	UPP1	7378	0,39	1,33	1,06	2407,9
Hs.288193	KPNA4	3840	0,24	0,03	1,05	753,1
Hs.355983	BZW1	9689	0,07	-0,51	1,05	2530,1
Hs.368157	PYGB	5834	0,18	1,16	1,05	187,1

Gene Identifiers			2log ratios HGF *versus* no HGF			overall raw expression
UniGene	Gene symbol	LocusLink	2 hours	8 hours	24 hours	EV
Hs.377028	C4.4A	27076	0,97	1,52	1,05	125,2
Hs.192221	ELL2	22936	0,78	-0,21	1,04	61,5
Hs.25155	NET1	10276	0,11	-0,49	1,04	212,8
Hs.435850	LYPLA1	10434	0,11	-0,28	1,04	1830,2
Hs.459072	LOC58489	58489	-0,13	0,68	1,04	227,9
Hs.489051	STEAP2	261729	0,14	-0,71	1,04	51,2
Hs.18676	SPRY2	10253	0,32	1,15	1,03	211,0
Hs.167165	FLJ12975	79867	-0,51	-0,58	1,03	30,8
Hs.244940	RDH10	157506	-0,08	0,18	1,03	163,8
Hs.6846	LPPR2	64748	-0,41	0,16	1,02	38,9
Hs.112405	S100A9	6280	0,00	0,00	1,02	26,3
Hs.117167	LOC283537	283537	-0,29	-0,35	1,02	100,2
Hs.433297	C10orf75	90271	-0,06	-0,20	1,02	64,0
Hs.493401	UHRF2	115426	-0,38	-0,39	1,02	416,5
Hs.513633	GPR56	9289	0,00	1,37	1,02	26,6
Hs.203691	DCBLD2	131566	-0,09	-0,71	1,02	283,7
Hs.44766	RP2	6102	0,20	-0,30	1,01	32,7
Hs.110642	NTSR1	4923	0,00	0,00	1,01	19,7
Hs.244723	CCNE1	898	0,30	1,05	1,01	113,7
Hs.336994	MTSS1	9788	-0,10	-0,42	1,01	13,6
Hs.485938	RRAGD	58528	-0,40	0,07	1,01	118,0
Hs.489722	ZNF277	11179	-0,45	-1,04	1,01	73,7
Hs.66	IL1RL1	9173	0,00	0,06	1,00	20,7
Hs.26608	C20orf100	84969	-0,13	1,43	1,00	119,6
Hs.80342	KRT15	3866	0,00	0,52	1,00	39,6
Hs.211933	COL13A1	1305	0,47	0,29	1,00	69,4
Hs.497723	MGC27165	283650	-0,75	1,28	1,00	14,8
Hs.515011	SMURF2	64750	0,10	-0,06	1,00	287,1
Hs.533635	KAB	9859	0,10	-0,68	1,00	375,0
Hs.213424	SFRP1	6422	0,17	0,57	0,99	72,2
Hs.98643	RAP2B	5912	0,07	0,39	0,99	287,7
Hs.502328	CD44	960	0,13	0,65	0,98	65,0
Hs.509909	NUMB	8650	0,29	0,27	0,97	111,4
Hs.128791	CGI-09	51605	0,30	0,08	0,97	364,6
Hs.459153	BNC1	646	0,72	0,15	0,97	101,8

Gene Identifiers			2log ratios HGF *versus* no HGF			overall raw expression
UniGene	Gene symbol	LocusLink	2 hours	8 hours	24 hours	EV
Hs.200770	SCAP2	8935	-0,27	-0,86	0,96	193,0
Hs.29692	FLJ36031	168455	0,54	0,85	0,96	77,8
Hs.153952	NT5E	4907	0,36	0,17	0,96	413,6
Hs.231295	PITPNC1	26207	-0,49	1,26	0,95	72,3
Hs.334873	CPM	1368	0,04	0,38	0,94	143,7
Hs.89714	CXCL5	6374	0,27	-0,66	0,93	607,3
Hs.281348	CCNJ	54619	0,73	-1,08	0,92	36,0
Hs.13680	AK092922	440426	0,35	0,34	0,92	172,9
Hs.59214	DNAJC3	5611	-0,19	-1,07	0,91	62,0
Hs.200250	CREM	1390	0,41	0,05	0,91	46,3
Hs.518857	SMARCA5	8467	-0,20	-1,13	0,91	523,0
Hs.525704	JUN	3725	0,99	0,91	0,90	193,6
Hs.269857	HRB2	11103	-0,02	-0,63	0,89	292,8
Hs.335079	MAP1B	4131	0,16	-1,82	0,89	314,8
Hs.434059	ETV4	2118	-0,25	1,03	0,89	145,8
Hs.467740	LPIN1	23175	0,46	-0,05	0,87	228,9
Hs.468426	SOCS5	9655	-0,05	-0,83	0,86	150,9
Hs.495138	MAPKAP1	79109	0,13	0,42	0,86	294,2
Hs.93667	C10orf78	119392	-0,39	-0,93	0,85	334,9
Hs.115166	SCEL	8796	-0,21	-0,73	0,84	74,5
Hs.227777	PTP4A1	7803	0,18	-1,24	0,84	590,8
Hs.532826	MCL1	4170	0,78	0,02	0,83	730,2
Hs.419195	FLJ35954	166968	-0,20	-1,30	0,82	56,1
Hs.208267	B3GNT5	84002	0,53	-1,02	0,82	91,4
Hs.514500	MGC29814	283991	-0,25	1,16	0,82	211,2
Hs.6790	DNAJB9	4189	-0,18	-0,64	0,80	92,2
Hs.91389	LOC169932		0,12	-0,70	0,80	73,8
Hs.133183	LOC284591	284591	0,05	0,02	0,80	53,2
Hs.287362	TLE3	7090	0,56	0,98	0,79	44,8
Hs.82101	PHLDA1	22822	1,30	0,57	0,79	719,7
Hs.144795	KCNMA1	3778	-0,16	0,11	0,79	58,7
Hs.532265	DKFZP564O0463	25879	-0,16	-0,82	0,78	533,6
Hs.375684	RAD18	56852	-0,07	-0,58	0,78	87,5
Hs.173484	LRRC8	56262	0,68	0,96	0,77	444,1
Hs.213467	TNFRSF10D	8793	0,34	-0,02	0,76	199,4

Gene Identifiers			2log ratios HGF *versus* no HGF			overall raw expression
UniGene	Gene symbol	LocusLink	2 hours	8 hours	24 hours	EV
Hs.434269	ARNTL2	56938	-0,42	-0,47	0,75	72,1
Hs.268742	C13orf12	51371	0,10	0,34	0,74	1929,6
Hs.469723	CHC1	1104	-0,11	-0,10	0,74	198,4
Hs.530791	LOC388114	388114	0,17	0,30	0,74	108,2
Hs.357901	SOX4	6659	-0,61	-0,09	0,73	39,2
Hs.435981	ERCC1	2067	0,01	1,51	0,73	298,7
Hs.196983	SSFA2	6744	0,25	-0,49	0,73	111,1
Hs.235069	RECQL	5965	-0,30	-1,26	0,72	194,0
Hs.369052	SELT	51714	-0,35	-1,51	0,71	366,4
Hs.477009	USP24	23358	-0,25	-1,14	0,71	134,2
Hs.178357	C9orf82	79886	0,22	-0,19	0,70	154,1
Hs.48343	MGC26963	166929	0,26	-0,90	0,68	106,3
Hs.103915	JMJD3	23135	1,57	1,06	0,68	95,5
Hs.118722	FUT8	2530	-0,05	-0,49	0,68	53,0
Hs.20084	RXRA	6256	-0,27	0,70	0,68	112,0
Hs.533468	FAM29A	54801	-0,15	-1,11	0,67	166,7
Hs.85962	HAS3	3038	0,18	0,72	0,67	413,4
Hs.472010	PRNP	5621	-0,07	-0,42	0,65	917,5
Hs.28491	SAT	6303	0,54	-0,02	0,64	848,0
Hs.509545	PBX2	5089	0,36	-0,09	0,64	111,5
Hs.520506	FBXO5	26271	-0,13	-0,75	0,63	244,1
Hs.497822	DUSP10	11221	0,19	0,38	0,61	49,8
Hs.512932	FLJ13213	79811	-0,06	-0,36	0,61	150,6
Hs.440049	MGC11349	79364	0,55	0,08	0,59	29,1
Hs.269092	CDYL	9425	0,05	0,09	0,58	328,1
Hs.406266	HK2	3099	0,25	0,56	0,58	140,5
Hs.209431	MGC3794	261726	-0,25	-0,71	0,57	154,2
Hs.516543	AGPS	8540	-0,16	-1,07	0,56	158,6
Hs.501513	CGI-37	51388	0,39	0,14	0,55	659,5
Hs.522590	EIF1AX	1964	-0,06	-1,08	0,54	504,9
Hs.510301	hmd2a08m3		-0,26	-0,33	-0,50	94,7
Hs.435255	UBXD1	80700	0,03	0,71	-0,52	206,5
Hs.482034	FLJ21657	64417	0,06	-1,59	-0,53	186,9
Hs.471873	DTYMK	1841	-0,13	0,55	-0,53	293,5
Hs.516697	MR-1	25953	-0,11	0,29	-0,55	126,1

Gene Identifiers			2log ratios HGF *versus* no HGF			overall raw expression
UniGene	Gene symbol	LocusLink	2 hours	8 hours	24 hours	EV
Hs.269512	FSTL1	11167	-0,18	-0,69	-0,56	267,5
Hs.414099	TNRC5	10695	-0,05	0,51	-0,56	103,5
Hs.410970	MYL5	4636	-0,26	-0,18	-0,57	40,9
Hs.516087	TEX261	113419	-0,23	0,12	-0,57	328,3
Hs.512894	TRPM7	54822	0,10	-1,03	-0,57	31,2
Hs.520189	ELOVL5	60481	0,20	-0,26	-0,57	684,4
Hs.461086	CDH1	999	0,23	-0,39	-0,58	405,6
Hs.437008	EPHB4	2050	0,30	0,26	-0,62	114,7
Hs.500775	ZNF207	7756	0,18	-0,86	-0,62	472,1
Hs.505004	TCEA2	6919	-0,22	0,62	-0,64	106,6
Hs.376681	MFN2	9927	0,18	0,31	-0,65	165,2
Hs.435126	SLC2A4RG	56731	-0,05	0,29	-0,65	130,8
Hs.414028	C9orf116	138162	-0,32	0,17	-0,66	53,8
Hs.175322	USP13	8975	-0,13	-0,77	-0,66	171,2
Hs.100009	CDK3	1018	-0,55	-0,11	-0,67	52,1
Hs.274408	CASKIN2	57513	-0,36	0,07	-0,67	32,9
Hs.536037	IMAGE 6191529		-0,06	-1,37	-0,68	56,3
Hs.433995	FLJ26120		1,10	-1,00	-0,70	123,6
Hs.369554	SLC16A5	9121	0,13	0,33	-0,70	139,7
Hs.375108	CD24	934	0,06	-1,14	-0,71	484,8
Hs.515003	C19orf6	91304	0,20	-0,16	-0,71	60,1
Hs.535030	MGC13098	84820	0,11	-0,74	-0,71	65,7
Hs.334305	DGAT2	84649	0,20	0,52	-0,72	79,5
Hs.269654	HSMPP8	54737	0,10	-0,82	-0,72	52,2
Hs.370510	IGSF4	23705	0,33	-0,81	-0,73	64,7
Hs.471528	C2orf33	56947	-0,29	-0,53	-0,78	1314,1
Hs.517366	LSS	4047	-0,08	0,34	-0,78	161,8
Hs.334534	GNS	2799	0,09	-0,38	-0,78	409,1
Hs.517666	DIA1	1727	-0,09	0,46	-0,78	1274,8
Hs.463511	H6PD	9563	0,11	0,48	-0,84	41,2
Hs.472101	PLCB4	5332	-0,33	-1,89	-0,84	63,1
Hs.204891	IL11RA	3590	-0,35	0,40	-0,85	65,3
Hs.524804	LOC338799	338799	-0,37	0,18	-0,85	47,4
Hs.143736	C20orf108	116151	-0,22	-0,98	-0,86	141,0
Hs.122575	EDG4	9170	-0,10	1,16	-0,86	72,7

Gene Identifiers			2log ratios HGF *versus* no HGF			overall raw expression
UniGene	Gene symbol	LocusLink	2 hours	8 hours	24 hours	EV
Hs.503998	TAGLN	6876	0,63	1,36	-0,88	89,5
Hs.110571	GADD45B	4616	-0,61	-0,55	-0,89	231,8
Hs.75798	C20orf111	51526	0,20	-0,52	-0,90	262,1
Hs.18376	CGN	57530	-0,18	-0,66	-0,91	88,1
Hs.165859	ANTXR1	84168	0,06	-0,44	-0,91	131,3
Hs.157180	IPP	3652	-0,29	-0,27	-0,93	44,0
Hs.142912	FZD2	2535	-0,66	-0,22	-0,94	148,0
Hs.335163	KIAA1102	22998	-0,09	-1,29	-0,94	55,4
Hs.472471	C20orf178	128866	0,06	0,21	-0,95	482,5
Hs.408312	TP53	7157	-0,29	0,22	-0,96	252,0
Hs.293970	ALDH6A1	4329	0,18	-0,28	-0,97	81,9
Hs.423103	HCFC1R1	54985	-0,08	0,52	-0,98	184,9
Hs.105891	TBC1D3	84218	0,11	-1,36	-1,00	56,4
Hs.292375	CHST13	166012	0,12	0,24	-1,00	26,0
Hs.482390	TGFBR3	7049	-0,42	-1,28	-1,01	53,0
Hs.90756	LOC152831	152831	-0,16	-1,29	-1,01	45,7
Hs.512029	B3GNTL1	284208	-0,72	0,19	-1,01	32,8
Hs.448468	NEK8	284086	-0,03	-0,64	-1,02	58,1
Hs.446352	ERBB2	2064	0,12	0,36	-1,03	95,7
Hs.148685	GPRC5B	51704	-0,25	0,34	-1,04	27,3
Hs.429434	GAB2	9846	-0,48	-0,56	-1,04	13,9
Hs.532277	ZNF250	58500	-0,52	-0,06	-1,04	44,1
Hs.463421	ABCC3	8714	0,37	-0,37	-1,05	188,6
Hs.25338	PRSS23	11098	-0,05	-0,74	-1,05	596,6
Hs.414396	PERQ1	64599	-0,26	0,00	-1,05	21,3
Hs.513100	ZSCAN2	54993	-0,10	0,04	-1,05	24,1
Hs.520814	TENS1	64759	-0,09	-0,52	-1,05	470,4
Hs.530792	GCHFR	2644	0,03	0,39	-1,05	284,2
Hs.80642	STAT4	6775	-0,09	-1,66	-1,06	83,2
Hs.134012	C1QL1	10882	-0,77	0,99	-1,06	96,8
Hs.408062	KNSL8	89953	-0,70	-0,59	-1,06	29,8
Hs.194679	WISP2	8839	0,05	-0,28	-1,07	62,4
Hs.164226	THBS1	7057	0,32	-0,96	-1,08	136,1
Hs.185807	COG7	91949	-0,23	0,21	-1,08	71,3
Hs.422889	NUDT6	11162	-0,57	-0,04	-1,08	82,2

Gene Identifiers			2log ratios HGF *versus* no HGF			overall raw expression
UniGene	Gene symbol	LocusLink	2 hours	8 hours	24 hours	EV
Hs.546389	YBX2	51087	0,00	0,56	-1,09	47,8
Hs.476319	FLJ10948	55268	0,51	0,18	-1,10	46,0
Hs.196177	PHKG2	5261	-0,26	0,08	-1,10	108,7
Hs.473256	LAMA5	3911	0,05	-0,33	-1,10	51,5
Hs.475055	BIK	638	0,03	0,01	-1,10	56,8
Hs.517586	MB	4151	0,13	1,13	-1,10	96,9
Hs.523789	TncRNA	283131	0,69	-1,97	-1,11	144,1
Hs.10136	BPHL	670	-0,33	0,54	-1,11	178,5
Hs.129126	MGC10992	92922	-0,22	0,69	-1,11	77,6
Hs.303197	BCL7C	9274	-0,07	0,63	-1,11	536,3
Hs.502745	FADS2	9415	-0,14	1,28	-1,11	180,7
Hs.516769	EFHD1	80303	-0,01	0,10	-1,11	43,7
Hs.127897	RAPGEF1	2889	0,12	0,33	-1,12	59,6
Hs.516243	TSRC1	54507	0,00	0,85	-1,12	47,5
Hs.328232	GPC1	2817	0,20	0,69	-1,13	277,4
Hs.306834	FKBP1B	2281	-0,14	0,65	-1,13	49,1
Hs.484047	KIBRA	23286	0,14	-0,19	-1,15	72,4
Hs.31130	TM7SF2	7108	-0,62	0,49	-1,15	219,6
Hs.194411	RAD52B	201299	-0,14	0,19	-1,15	117,5
Hs.477375	MYLK	4638	-0,03	0,12	-1,18	78,5
Hs.158748	SLC35F3	148641	-0,17	-0,29	-1,18	27,1
Hs.438419	SLC29A3	55315	-0,25	0,43	-1,18	69,2
Hs.381081	PSMB9	5698	-0,34	0,30	-1,19	844,0
Hs.128041	ATP10A	57194	0,01	-0,53	-1,20	36,2
Hs.89603	MUC1	4582	-0,17	0,77	-1,21	292,3
Hs.504908	LMO3	55885	-0,49	-1,80	-1,22	179,8
Hs.546384	GMPPB	29925	-0,24	0,99	-1,22	151,7
Hs.434828	HES7	84667	0,82	0,87	-1,23	56,5
Hs.19545	FZD4	8322	-0,17	-0,84	-1,24	56,6
Hs.131810	FLJ35976	284618	0,52	-1,41	-1,25	58,1
Hs.70327	CRIP1	1396	-0,16	0,65	-1,26	1498,8
Hs.111903	FCGRT	2217	0,51	0,69	-1,26	58,6
Hs.467545	HSPG2	3339	0,02	-0,07	-1,26	62,2
Hs.524790	CS0DD005YE10		0,03	-0,09	-1,26	120,0
Hs.98669	ADPRHL1	113622	-0,20	0,50	-1,27	54,5

Gene Identifiers			2log ratios HGF *versus* no HGF			overall raw expression
UniGene	Gene symbol	LocusLink	2 hours	8 hours	24 hours	EV
Hs.1162	HLA-DMB	3109	-0,54	-0,16	-1,28	69,8
Hs.517070	SLPI	6590	-0,04	-0,01	-1,28	159,7
Hs.546985	DKFZp762C074		0,00	-1,24	-1,29	35,4
Hs.458573	PDGFRL	5157	0,03	0,63	-1,30	69,3
Hs.12106	MMAB	326625	-0,34	0,60	-1,32	100,1
Hs.463110	ANXA8	244	0,24	-0,89	-1,33	64,4
Hs.112423	DKFZp586I1420	222161	-0,40	-1,64	-1,34	44,3
Hs.26530	SDPR	8436	-0,50	-1,58	-1,36	202,8
Hs.527524	KIAA1280	55841	-0,19	-0,22	-1,36	38,9
Hs.387251	EVI5	7813	-0,75	0,30	-1,38	45,4
Hs.140978	LOC144501	144501	-0,33	-0,23	-1,41	212,1
Hs.546392	PLAC8	51316	0,00	-0,30	-1,48	161,0
Hs.76224	EFEMP1	2202	-0,09	-0,73	-1,51	188,3
Hs.502989	UNC93B1	81622	-0,06	0,53	-1,51	85,8
Hs.481879	FLJ27323		0,35	-1,18	-1,52	156,1
Hs.3797	RAB26	25837	0,26	-0,66	-1,56	116,6
Hs.447901	RHOV	171177	-0,30	-0,45	-1,57	420,6
Hs.440829	CEBPD	1052	-0,18	-0,19	-1,65	140,4
Hs.523841	ALDH3B1	221	-0,11	-0,20	-1,66	128,3
Hs.146978	GPR64	10149	-0,29	-1,14	-1,69	133,1
Hs.147895	ZNF518	9849	0,48	-1,97	-2,00	60,9
Hs.532596	FLJ23566		-0,26	-1,50	-2,00	192,7
Hs.73708	ANKRD2	26287	-0,22	0,21	-2,31	108,1
